# Novel *SFRP2* DNA Methylation Profile Following Neoadjuvant Therapy in Colorectal Cancer Patients with Different Grades of BMI

**DOI:** 10.3390/jcm8071041

**Published:** 2019-07-17

**Authors:** Amanda Cabrera-Mulero, Ana B. Crujeiras, Andrea G. Izquierdo, Esperanza Torres, Duncan Ayers, Felipe F. Casanueva, Francisco J. Tinahones, Sonsoles Morcillo, Manuel Macias-Gonzalez

**Affiliations:** 1 Department of Endocrinology and Nutrition, Virgen de la Victoria University Hospital, University of Malaga (IBIMA), 29010 Málaga, Spain; 2 CIBEROBN (CIBER in Physiopathology of Obesity and Nutrition CB06/03/0018), “Instituto de Salud Carlos III”, 28029 Madrid, Spain; 3 Epigenomics in Endocrinology and Nutrition Group, Instituto de Investigación Sanitaria (IDIS), Complejo Hospitalario Universitario de Santiago (CHUS/SERGAS), 15706 Santiago de Compostela, Spain; 4 Unidad de Gestión Clínica de Oncología Intercentros Hospital Universitario Virgen de la Victoria, 29010 Málaga, Spain; 5 Centre for Molecular Medicine and Biobanking, University of Malta, 2080 Msida MSD, Malta; 6 Faculty of Biology, Medicine and Health, The University of Manchester, Manchester M1 7DN, UK; 7 Laboratorio Investigación Biomédica 1ª Planta, Hospital Universitario Virgen de la Victoria, Campus de Teatinos s/n 29010, 29010 Málaga, Spain

**Keywords:** *SRFP2*, DNA methylation, BMI, colorectal cancer, therapy, location

## Abstract

The relationship between body weight and different cancers is now well-recognized and among such cancers, colorectal cancer (CRC) is reported most frequently. Our group recently published findings, through an epigenome-wide association study, suggesting that body mass index (BMI) could act as a relevant risk factor in the CRC. In addition, aberrant *SFRP2* methylation is one of the major mechanisms for Wnt signaling activation in CRC. Conversely, neoadjuvant chemo-radiotherapy appears to alter the rectal cancer epigenome. This study was aimed to evaluate the effect of obesity, measured by BMI, on the methylation of *SFRP2* in tumor samples of patients with CRC. Non-treated CRC patients and CRC patients treated with pre-operative neoadjuvant therapy from 2011 to 2013 were included and classified by BMI < 25.0 kg/m^2^ and BMI > 25.0 kg/m^2^. *SFRP2* DNA methylation in tumor samples was measured by pyrosequencing. Our findings suggest a possible interaction between *SFRP2* methylation levels and BMI in CRC tumor samples. The correlation of *SFRP2* hypomethylation with an elevated BMI was stronger within the non-treated CRC patient group than within the treated CRC patient group. We have successfully demonstrated that the beneficial association of tumor *SFRP2* hypomethylation is dependent on patient BMI in non-treated CRC, suggesting a possible tumor suppressor role for *SFRP2* in overweight and obese patients. Additional studies of clinical pathologies would be necessary to strengthen these preliminary results.

## 1. Introduction

One of the biggest challenges facing biomedical science is finding promising diagnostic, prognostic, and predictive biomarkers of non-communicable diseases of multi-factorial origin, such as colorectal cancer (CRC). Based on global cancer statistics, CRC is one of the leading causes of mortality from all cancers and the cancer-related mortality has increased by almost 40% in the last 40 years [1]. Consequently, novel promising biomarkers are required to increase the CRC screening sensitivity and predict more effective responses to individual cancer therapies.

DNA and miRNAs methylation of tumor suppressor genes and oncogenes is a key process in human carcinogenesis [2,3]. Moreover, epigenetic changes of tumor suppressor genes are a recognized factor in Knudson´s model of cancer [4]. According to the literature, epigenetic remodeling of several genes (*KRAS, APC, p53, DCC,* genes of the Rho family of GTPases, *MACC1, Met, MTA1, RASSF1A, SFRP1*, etc.) are associated with malignant cellular transformation, progression, invasion, and metastasis in colorectal cancers [5,6,7]. Consequently, aberrant DNA methylation also offers an opportunity for its exploitation as ideal biomarkers for CRC prediction, diagnosis, and prognosis [8]. Furthermore, the identification and validation of several hyper- and hypomethylated genes in CRC can be highly useful for employment as novel biomarkers for tumor development. Previous studies in CRC observed that *LINE-1* (long interspersed nucleotide element) hypomethylation was associated with more advanced disease stages [9,10]. 

One important example of such key molecular players is the secreted frizzled-related protein type 2 (*SFRP2*), which can be deemed as a potential epigenetic biomarker of CRC. Moreover, *SFRP2* (a Wnt signaling inhibitor) is hypermethylated in CRC [11,12,13]. Recently, a number of studies demonstrated that *SFRP2* is not only hypermethylated in CRC tissue, but also in a spectrum of biological tissues including blood and stools, suggesting that *SFRP2* methylation might be a potential non-invasive biomarker for CRC screening [14,15,16]. 

Currently, it is well established that excess body weight, defined by the body mass index (BMI), can be considered to be a risk factor for the incidence and progression of CRC [17]. The underlying mechanisms linking obesity to CRC are still a matter of debate. However, BMI-associated DNA methylation profile changes could provide one of the missing links between obesity and CRC [18]. Recently, we have reported that the hyper-methylation of the *SFRP2* promoter exhibited a strong correlation with BMI in tumor samples, consequently suggesting adiposity as a prognostic factor in patients with CRC [14].

Presently, neoadjuvant chemo-radiotherapy, followed by radical surgery is considered to be the standard of care for patients with locally advanced rectal cancer [19]. Several authors have described how the patient characteristics, such as BMI and internal distribution of adipose tissue, have the potential to interfere with radiation dosing or delivery, leading to appreciable differences in treatment response and survival outcomes [20]. On the other hand, some studies have reported that DNA methylation could be a crucial factor that determines oncological treatment efficacy of, both, chemotherapy and radiotherapy [21]. However, it has been suggested that oncological treatment can also induce epigenetic changes that could mediate treatment response [22]. Ultimately, novel therapeutic approaches opt for utilizing adjuvant methyltransferase inhibitors, in order to increase treatment efficacy [23].

Considering the correlation between the *SFRP2* methylation status and BMI in CRC patients [14], together with the potential effect of neoadjuvant treatment on DNA methylation pattern; we propose that *SFRP2* methylation could be associated with specific clinical outcome of CRC patients and this relationship could be affected by BMI or treatment. In the present study, we have specifically focused on the analysis of tumor and non-tumor samples for the methylation status of *SFRP2* in an independent prospective cohort study, together with its association with the clinical and pathological features of CRC patients.

## 2. Materials and Methods

### 2.1. Study Population

A total of 75 CRC patients diagnosed by biopsy or colonoscopy were included in the present study from Virgen de la Victoria University Hospital (Málaga, Spain), recruited from October 2011 to September 2013. One cohort group consisted of 53 overweight or obese patients (BMI > 25 kg/m^2^), while 22 were non-obese participants (BMI < 25 kg/m^2^). All participants underwent surgery with curative intention, by hemicolectomy, lower anterior resection with ileostomy (caused by a carcinoma of the CRC), followed by a total mesocolorectal excision. A formalin-fixed paraffin-embedded (FFPE) (10 sections of 14 μm) tumor area and an adjacent tumor-free area were obtained from all CRC patients. 

Participants included in the study had primary CRC whose medical records/pathological examinations were complete. The exclusion criteria were patients with inflammatory bowel disease (Crohn´s disease or ulcerative colitis) and patients who had evidence of hereditary non-polyposis colorectal cancer or familial adenomatous polyposis. All participants were anonymized and gave their written informed consent. The study was performed in accordance with the Declaration of Helsinki and was approved by the Ethics Committees of Virgen de la Victoria University Hospital (registration number 0311/PI7) (Málaga, Spain).

The clinical–pathological parameters of the cohort groups were confirmed by reviewing the patient medical records and pathology files. The pathological diagnosis, tumor histology, and staging were performed according to the Classification of the “World Health Organization Classification of Tumours of the Digestive System” (2016) [24]. Rectal cancer patients were treated according to local protocols, after a routine workout, including a turaco abdominal scan and a pelvis MRI. Those classified as T3–4 or N 1–3 were proposed for neoadjuvant chemoradiation treatment with pelvic radiotherapy 50Gy (2 Gy/fraction) and concomitant administration of fluoropyrimidine-based chemotherapy, followed by total mesorectal excision in 6–8 weeks. Patients were sub-classified as treated (those that were designated for neoadjuvant therapy), while those who did not receive any pre-operative treatment were classified as non-treated.

### 2.2. Biochemical Determination

Fasting blood samples were obtained from the antecubital vein and placed in vacutainer tubes (BD Vacutainer™, Franklin Lakes, NJ, USA). Serum glucose, total cholesterol, triglycerides, and HDL cholesterol (HDL-c) were measured in a Dimension auto analyzer (Dade Behring Inc., Deerfield, IL, USA) through enzymatic methods (Randox Laboratories Ltd., Crumlin, UK). The LDL cholesterol (LDL-c) was calculated using the Friedewald equation [25]. Insulin was quantified by radioimmunoassay (BioSource International, Camarillo, CA, USA). Corrected calcium was assessed using a complex metric method from Boehringer Hitachi 717. Alkaline phosphatase was calculated using ELISA (LifeSpan Biosciences Inc., Madrid, Spain). Insulin growth factor type 1 (IGF-1) was determined using Human IGF1 ELISA Kit (Abcam, Madrid, Spain). The homeostasis model assessment of insulin resistance (HOMA-IR) was calculated using the following equation: HOMA-IR = fasting insulin (IU/mL) x fasting glucose (mmol/L)/22.5 [26].

### 2.3. Human Colorectal CarcinomaCell Lines and SFRP2 Expression Assay by RT-qPCR

HTC116 cell line (*Homo Sapiens* colon colorectal carcinoma, ATCC, Manassas, VA, USA), LoVo cell line (*Homo Sapiens* colorectal adenocarcinoma derived from metastatic site—left supraclavicular region, ATCC, Manassas, VA, USA) and Caco-2 cell line (*Homo Sapiens* colon colorectal adenocarcinoma, ATCC, Manassas, VA, USA) were used to study the gene expression of *SFRP2*. Cells were cultured in Dulbecco’s modified Eagle’s medium containing 10% fetal bovine serum, penicillin, and streptomycin at 37 °C and 5% CO_2_. Cell lines were treated with the DNA demethylation agent 5-aza-2′-deoxycitydine (AZA) (A3656, Sigma Aldrich, Madrid, Spain) at 5μM for 72 hours.

Consequently, RNA from cell lines was isolated using QiampRNA Mini Kit (Qiagen GmbH, Hilden, Germany), according to the manufacturer’s instructions. The purity and concentration of the RNA was determined by the 260/280 absorbance ratio using the Nanodrop 2000 platform (Thermo scientific, USA). For cDNA synthesis, a fixed amount of 1 µg of the total RNA was reverse transcribed using random hexamers as primers and Moloney Murine Leukemia Virus reverse transcriptase (Roche Diagnostic, Rotkreuz, Switzerland). Gene expression was assessed by real-time PCR, using an Applied Biosystems 7500 Fast Real-Time PCR System (Applied Biosystems, Darmstadt, Germany) with TaqMan technology. The reaction was performed following the manufacturer's protocol of TaqMan technologies Premix Ex Taq™ (Probe qPCR) (Takara, Madrid, Spain), in a final volume of 12.5 µL. The cycle program consisted of an initial denaturing of 20 s at 95 °C, then 45 cycles of a 3 s denaturing phase at 95 °C, followed by a 30 s annealing period. The commercially available and revalidated TaqMan primer/probe sets used in our samples were *SFRP2* and *PPIA* TaqMan Probes (IDT technologies, Madrid, Spain). A threshold cycle (Ct value) was obtained for each amplification curve and a ΔCt value was first calculated by subtracting the Ct value for Cyclophilin A cDNA from the Ct value for each sample and transcript. Fold changes compared with the endogenous control were then determined by calculating 2^−ΔCt^, and relative quantification results were obtained.

### 2.4. DNA Extraction, Bisulfite Treatment, and Pyrosequencing

Total genomic DNA from 75 CRC paraffin samples (formalin-fixed paraffin embedded (FFPE) (10 sections of 14 μm) was isolated using the QIAmp DNA FFPE Tissue Kit (Qiagen GmbH, Hilden, Germany), with a xylene wash, to remove the paraffin. DNA concentration and quality was determined using NanoDrop 2000 (Thermo scientific, USA). DNA methylation analyses were performed using bisulfite-treated DNA, followed by a highly quantitative analysis, based on PCR-based pyrosequencing. The bisulfite conversion was conducted with 2 μg of genomic DNA isolated from each sample, using Epitect Bisulfite conversion, according to the manufacturer´s instructions (Qiagen GmbH, Hilden, Germany). Consequently, the *SFRP2* promoter and *LINE-1* amplification steps were performed in a total volume of 25 μL, with a starting primer concentration of 10 μM (*SFRP2*: Primer 5′3′ forward and reverse respectively: TTTGATTTTTTTAYGGTATTGGGGAGTA and ATAAAACCCRAAACCTACCC. *LINE-1*: Primer 5′3′ forward and reverse respectively: TAGGGAGTGTTAGATAGTGG and AACTCCCTAACCCCTTAC). The reverse primer was biotinylated, in order to purify the final PCR product, using sepharose beads. Finally, 20 μL of the PCR products were pyro sequenced using the PyroMarkTMQ96 ID Pyrosequencing System, using a 0.4 μM sequencing primer. The primer sequences used in this analysis were designed using Qiagen’s PyroMark Assay Design 2.0 software (Qiagen, Hilden, Germany).

The methylation level was expressed as the percentage of methylated cytosine over the sum of methylated and unmethylated cytosines. Non-CpG cytosine residues were used as built-in controls to verify bisulfite conversion. The values are expressed as the mean for all the sites and individually for seven CpGs at the *SFRP2* gene promoter and for six CpGs at the *LINE-1* sequence [27,28]. We also included unmethylated and methylated DNA as controls in each run (New England Biolabs, Ipswich, MA, USA).

### 2.5. Statistical Analysis

The results were expressed as the mean ± standard deviation (or percentage) where appropriate. Student’s *t* test was used for comparing anthropometric and biochemical data between non-obese and overweight/obese groups. Paired *t*-test was performed to compare means between tumor and tumor-free area. Student’s *t* test and Mann–Whitney U-test were used for comparisons of *LINE-1* and SFRP2 methylation between study groups. Spearman’s correlation analyses were performed to study the correlations between *LINE-1* and *SFRP2* methylation. A regression linear model was used to perform multivariate analysis. All analyses were performed using the R statistical software, version 2.8.1 (Department of Statistics, University of Auckland, Auckland, NZ; http://www.rproject.org/).

## 3. Results

### 3.1. Clinical, Anthropometrical, and Biochemical Characteristics of the CRC Patients

There were no differences in anthropometric and biochemical variables between subjects with BMI < 25 or BMI > 25, except for the BMI itself (as expected) and the insulin levels (Table 1).

### 3.2. Methylation Status of SFRP2 in CRC Tissue

**Figure 1:**
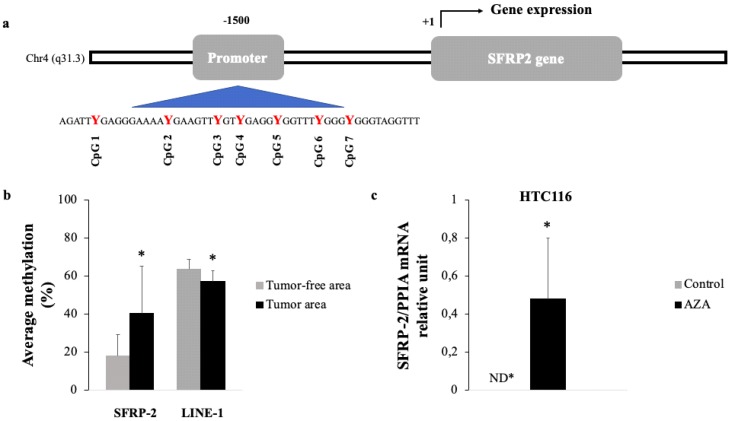
Methylation status of *SFRP2* in tumoral tissue of patients with CRC. (**a**) A representative picture of the genomic structure of the human *SFRP2* gene on chromosome 4q31 and the CpG island included in this study. Bioinformatic analysis demonstrated seven CpG-rich islands located at the −1500 position, relating to the transcription start site (+1). (**b**) Methylation levels of the *SFRP2* and *LINE-1* genes in 75 tumor tissue biopsies of CRC and adjacent tumor-free areas were analyzed by bisulfite treatment and pyrosequencing. Comparison through paired *t*-test (**c**) Quantitative RT-PCR was used to determine the expression of *SFRP2* in HTC116, after treatment with AZA, during 72 h. The mRNA expression of *SFRP2* was normalized to the *PPIA* expression. The results are displayed as mRNA relative mean expression ± SD. Abbreviations: *SFRP2*—secreted frizzled-related protein type 2; *LINE-1*—long interspersed element type 1; *PPIA*—peptidylprolyl Isomerase A; HTC116—*Homo sapiens* colon colorectal carcinoma; AZA—5-aza-2′-deoxycytidine; ND—not detected.

First of all, we initiated our analysis based on previous data of the *SFRP2* promoter performed and published by our group and obtained from the Infinium Human Methylation 450 BeadChip array [14].The study sequence of the *SFRP2* promoter used in our analysis showed seven CpG sites in the position −1500, relative to the expected transcription start site (+1),which was consequently utilized to determine the methylation status of the *SFRP2* promoter (Figure 1a). 

We aimed to evaluate the *SFRP2* methylation status in both CRC tissue and adjacent tumor-free tissue from 75 CRC patients, by pyrosequencing after bisulfite treatment.

The results revealed hyper-methylation of the *SFRP2* promoter, since *SFRP2* methylation in tumor tissue was significantly higher (40.30%)in comparison to the levels obtained for adjacent tumor-free tissue areas (17.86%). Conversely, *LINE-1* methylation was significantly decreased in the tumor area (57.38%), when compared to the tumor-free area (63.50%) (Figure 1b).

In addition, to test whether the hyper-methylation of *SFRP2* promoter is functionally associated with a down-regulated expression of *SFRP2* at the transcriptomic level, we performed in vitro demethylation assays, using different colorectal carcinoma cell lines.

Accordingly, we employed RT-qPCR assays to evaluate whether *SFRP2* mRNA is differentially expressed, before and after treatment, with a methylation inhibitor. First, we treated cell lines for 72 h with 5 μM of AZA, a methylation inhibitor. Consequently, we quantified the mRNA expression of *SFRP2* through RT-qPCR. The HTC116 control showed undetectable mRNA expression of *SFRP2*, while the HCT116 treated with AZA resulted in a significant and clear increase of mRNA *SFRP2* expression, as shown in Figure 1c. However, despite measuring the *SFRP2* gene expression in three different human colorectal carcinoma cell lines (HCT116, LoVo, and Caco-2), we only observed expression recovery of *SFRP2* in HCT116, after the AZA treatment. The lack of results from the other cell lines could be explained by their inherent biological heterogeneity from these cell lines.

### 3.3. Baseline Characteristics of Colorectal Cancer Patients with Regards to their BMI Category

Clinical, pathological, and tumor molecular features according to the BMI in proximal colon cancer (cecum, ascending colon, and transverse colon), distal colon cancer (descending colon, and sigmoid colon), and rectal colon cancer are summarized in Table 2.

We observed that there were no differences between the two groups for any of the oncological variables studied.

### 3.4. Methylation of SFRP2 in CRC Tissue is Associated with BMI

**Figure 2:**
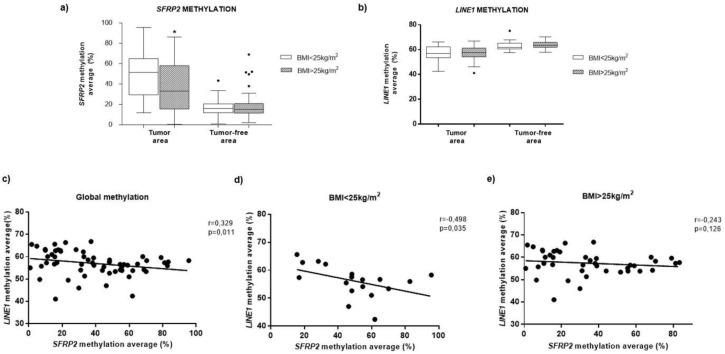
Relationship between BMI and *SFRP2* methylation in colorectal cancer. Methylation levels of the *SFRP2* (**a**) and *LINE-1* (**b**) genes, in 75 tumor tissue biopsies of CRC and adjacent tumor-free areas, in BMI < 25 and BMI > 25 kg/m^2^, analyzed by Mann–Whitney U-test. (**c**) Correlations between global methylation in *SFRP2* and LINE-1 genes in all subjects (**d**) in individuals with BMI < 25 kg/m^2^ and (**e**) those with BMI > 25 kg/m^2^ subjects. Abbreviations: *SFRP2*—Secreted frizzled-related protein type 2; *LINE-1*—Long interspersed element type 1; BMI—body mass index.

In order to investigate whether adiposity affects DNA methylation levels, the methylation pattern of *SFRP2* and *LINE-1* was compared in tumor tissue and tumor-free area, in both BMI groups. Our results showed that the *SFRP2* methylation was significantly lower in overweight/obese individuals (34.06%), compared to the non-obese individuals (51.24%) (Figure 2a), while *LINE-1* methylation did not show any significant difference in BMI (56.68% for non-obese subjects and for overweight/obese individuals 57.69%) (Figure 2b). We also observed a significant negative correlation between global *LINE-1* and *SFRP2* methylation in CRC tumor tissue (*r* = −0.329, *p* = 0.011) (Figure 2c). However, when the subjects were divided according to the BMI, this association only remained significant in subjects with BMI < 25 (Figure 2d,e).

### 3.5. SFRP2Methylation in CRC Patients with and without Neoadjuvant Treatment

The specific location of the tumor is a factor to be considered, as they could present biological and clinical differences. We analyzed the methylation *SFRP2* status according to the tumor location. First of all, we observed that *SFRP2* methylation was significantly higher on the right-sided CRC (57.33%), as compared to the left-sided CRC (36.60%). However, in this classification, most patient tumor samples were derived from the rectum. 

**Figure 3:**
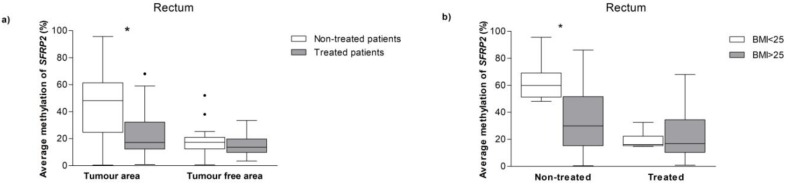
Analysis of abdominal location and therapy of CRC on the *SRFP2* methylation. Analysis of location and CRC patients treated on *SFRP2* methylation status. Methylation levels of the *SFRP2* divided by the treated versus the non-treated patients in areas with tumor and in tumor-free areas (**a**), and rectum separated by BMI in the treated versus the non-treated patients only in the samples with tumors (**b**). Mann-Whitney U-test was performed to compare the means. Abbreviations: *SFRP2*—Secreted frizzled-related protein type 2; BMI—body mass index.

Primary rectal cancer requires specific surgical treatment (total mesorectal excision, preceded by neoadjuvant radiotherapy or chemoradiotherapy); so we aimed to evaluate whether the neoadjuvant therapy could be affecting the methylation levels. Patients who received treatment displayed lower *SFRP2* methylation levels than those without treatment, within the tumor tissue, though not in the tumor-free area (Figure 3a). Finally, we assessed the *SFRP2* methylation status according to the BMI, in the treated and the non-treated patients. In the group of patients who received neoadjuvant therapy, there was no statistical difference in the *SFRP2* methylation levels regarding BMI. However, within the non-treated patients, those with BMI > 25 showed significantly lower *SFRP2* methylation levels, when compared to the BMI < 25 group (Figure 3b), albeit only in the tumor samples.

At last, to evaluate whether this association between *SFRP2* methylation and therapy could be due to other potential confounder variables, we performed a multivariable analysis, including age, BMI and presence of diabetes mellitus. We proved that neoadjuvant treatment was the main variable explaining the variability of *SFRP2* methylation levels (Table 3).

## 4. Discussion

Aberrant DNA methylation in gene promoters has been strongly associated with key dysregulations in the oncogenesis for most human tumor models, including colorectal cancer [29]. Presently, there is a large list of established genes subjected to abnormal DNA methylation that are consequently implicated in pivotal physiological processes, including tumor suppression, which can ultimately prove to be of essential utility as novel, epigenetics-based tumor biomarkers [30]. 

In this study, we evaluated the methylation status of *SFRP2*, which functionally acts as an inhibitor of Wnt signaling in CRC, together with BMI influences on such a methylation status. CRC is most commonly initiated by accumulation of β catenin in the Wnt signaling pathway, leading to the activation of the Wnt target genes [31]. 

However, contradicting results were also published in the scientific literature, stating that the modification of the prognostic impact of obesity by a positive status for nuclear β catenin was associated with a significantly improved survival rate for colorectal cancer [32]. 

The results of our study demonstrated that decreased *SFRP2* methylation in CRC was associated with an increased BMI. It has been established that adipose tissue is associated with local inflammation due to increased production of cytokines and pro-inflammatory factors through the macrophages present in adipose tissue [33]. This inflamed state was able to create a chronic low-grade inflammation that can activate the Wnt/β catenin pathway, where *SFRP2* plays an important regulatory role in the carcinogenesis process of CRC [34]. In essence, the functional role of *SFRP2* in CRC and its potential influence through an increased BMI, could explain, at least partially, the process through which obesity and Wnt/β catenin might exert their effect on carcinogenesis through independent molecular pathways [35].

The *SFRP2* methylation status was found to be associated with a decreased gene expression of *SFRP2* [36] and, furthermore, was found to be involved in carcinogenesis through down-regulation of tumor suppressor genes. Indeed, *SFRP2* can be detected in many biological samples, such as serum and stools, rendering it a potential non-invasive biomarker for many carcinogenic processes [37]. In this study we discovered that *SFRP2* was hypermethylated in CRC tumor tissue, when compared to adjacent tumor-free tissue areas. In addition, the hypermethylated *SFRP2* in vitro (as found in the HTC116 CRC tumoral cell line) were presumably associated with suppression of gene expression. Following treatment with AZA as a demethylating agent, the expression of *SFRP2* was significantly increased and restored. This suggests that *SFRP2* demethylation has a functional role in the transcriptional control of *SFRP2*. Our study was not focused on exploring the potential mechanisms involved in this decrease of DNA methylation pattern, although a possible explanation could be due to a de-regulation of DNA methyltransferase 1 (DNMT1). In most tumors, an altered regulation of this enzyme was described, which is involved in maintaining methylation patterns [38]. Studies have shown that DNMT inhibition promotes gene expression of *SFRP2*, suggesting DNMTs can modulate *SFRP2* methylation status [39].

Genome-wide DNA hypo-methylation of *LINE-1* is associated with a worse prognosis in early-stage colorectal cancer, though the majority of the performed studies did not reveal a relation between *LINE-1* hypomethylation and clinical outcome in more advanced disease stages [40]. However, our study results confirmed this association, since we revealed a low methylation rate for *LINE-1* in tumor samples, without any association or relationship with the clinical–pathological parameters of colorectal tumors.

Previous studies have reported that the location of tumors could influence treatment choice, whereby a large percentage of survival patients were specifically patients who presented primary CRC tumors on the left-side of the abdomen [41]. Consequently, we focused our study on *SFRP2* methylation in rectum tumor samples. Treated patients demonstrated a decrease of *SFRP2* methylation, independent of the BMI value. This supports the hypothesis that both *SFRP2* methylation and BMI could have a potential role in the development of CRC. Additionally, in the non-treated group, we observed higher *SFRP2* methylation levels in patients with low BMI, suggesting a possible tumor suppressor role of *SFRP2* in overweight/obese patients.

These results point to a possible interaction between BMI and neoadjuvant treatment. Moreover, recently, described epigenetic changes in DNA have also been underlined as mediators of the networking between obesity and cancer [42,43]. Li et al. reported that obesity led to epigenetic remodeling that resulted in differential gene expression related to metabolism and tumorigenesis. Moreover, this epigenetic signature could be reverted by weight loss reinforcing the role of BMI in the epigenetic mark of genes involved in neoplasia [44].

Limitations of this research include the lack of large cohort groups in order to analyze the effect of therapy with more statistical significance and perform survival studies. We could also not demonstrate the causal relation between methylation and gene expression, since expression data were obtained from human colon cancer cell lines. Finally, due to the challenge involved in obtaining high integrity RNA from tumor paraffin-embedded samples, we could not evaluate the gene expression from different DNA methyltransferases.

## 5. Conclusions

We demonstrated that the DNA methylation levels of *SFRP2* are lower in overweight/obese patients, in comparison with non-obese patients, specifically in tumor areas, supporting the hypothesis that BMI could play a protective role in the development of CRC. We also examined that the patients undergoing neoadjuvant treatment showed similar methylation values of *SFRP2*, independent of their BMI, suggesting that neoadjuvant treatment might have an effect on the epigenetic regulation of *SFRP2*, though further studies of clinical pathologies for such patients would be necessary to strengthen these preliminary results.

## Figures and Tables

**Table 1:** Baseline clinical, anthropometrical, and biochemical variables of the colorectal cancer (CRC) patients, according to the body mass index (BMI). Data are expressed as mean ± standard deviations or proportions.

Variables	BMI < 25 kg/m^2^ (*n* = 22)	BMI > 25 kg/m^2^ (*n* = 53)	*p*-Value
Age (years)	62.91 ± 11.21	66.64 ± 7.91	NS
Male/female	14/8	38/15	NS
BMI (kg/m^2^)	23.33 ± 1.32	29.20 ± 3.43	*p* < 0.05
Waist circumference (cm)	89.80 ± 12.01	101.39 ± 12.93	NS
Glucose (mg/dL)	122.57 ± 48.51	120.00 ± 40.40	NS
Insulin (µUI/mL)	4.07 ± 2.30	7.16 ± 5.63	*p* < 0.05
HOMA-IR	1.42 ± 1.38	2.25 ± 2.17	NS
Diabetes Mellitus (%)	30	29.2	NS
Triglycerides (mg/dL)	146.71 ± 76.36	171.92 ± 82.52	NS
Total cholesterol (mg/dL)	180.76 ± 39.90	169.13 ± 36.60	NS
HDL-c (mg/dL)	42.86 ± 15.07	39.51 ± 13.57	NS
LDL-c (mg/dL)	109.99 ± 30.94	100.72 ± 30.88	NS
Corrected calcium (mg/dL)	9.09±0.32	8.94 ± 0.54	NS
Alkaline phosphatase (U/L)	63.09 ± 26.76	72.57 ± 30.65	NS
IGF-1 (ng/mL)	137.26 ± 48.41	128.75 ± 73.92	NS

Abbreviations: BMI—Body mass index; NS—Non-significant; HOMA-IR—Homeostasis model assessment of insulin resistance; HDL-c—High density lipoprotein cholesterol; LDL-c—Low density lipoprotein cholesterol; IGF-1—Insulin growth factor type 1.

**Table 2:** Characteristics in CRC patients, locations, therapy, and stages. Comparisons were performed by chi-square test.

Variables	BMI < 25 kg/m^2^ (*n* = 22)	BMI > 25 kg/m^2^ (*n* = 53)	*p*-Value
Location			0.37
Proximal (Cecum, Ascending, Transverse)	2	12	
Distal (Descending, Sigmoid)	5	9	
Rectum	15	32	
Stage			0.43
I	6	13	
II	8	12	
III	7	20	
IV	1	8	
Treatments (previous surgery)			
Radiotherapy + Chemotherapy	4	13	0.25
Radiotherapy	2	1	
Chemotherapy	0	4	
Non-treatment	16	35	

Abbreviations: BMI—Body mass index; CRC—colorectal cancer.

**Table 3:** Multivariate regression analysis of *SFRP2* methylation levels according to the neoadjuvant therapy.

	*R*^2^ = 0.280		95% IC	
Variables	Standardized β	*p* Value	Lower Bound	Upper Bound
BMI (BMI < 25 vs. BMI > 25)	−0.339	0.022	−32.166	−2.700
Neoadjuvant therapy (treated vs. non treated)	−0.419	<0.001	−33.657	−5.860
Diabetes mellitus (diabetic vs. no diabetic)	−0.297	0.064	−34.535	1.013
Age (years)	0.188	0.218	−0.284	1.201

## Data Availability

Data will be not shared. An explicit statement was not included in the informed consent to allow data sharing. Data were not collected anonymously. Research data are stored with patients’ name, contact information, or other identifiers.

## Abbreviations

CRC	colorectal cancer
*SFRP2*	secreted frizzled-related protein 2
LINE-1	long Interspersed Element-1
BMI	body mass index
HDL-c	high density lipoprotein cholesterol
LDL-c	low density lipoprotein cholesterol
FFPE	formalin-fixed paraffin-embedded
